# No Hepatitis Delta Virus Seropositivity among Blood Donors with Overt and Occult Hepatitis B Infection in Dalian, Liaoning Province, China

**DOI:** 10.3390/v15071509

**Published:** 2023-07-06

**Authors:** Xuelian Deng, Dan Liu, Maelenn Pailine Delcourt, Huihui Gao, Lu Zhou, Daniel Candotti

**Affiliations:** 1 Dalian Blood Center, No. 90 Yan’an Road, Zhongshan District, Dalian 116001, China; 2 Department of Virology, Henri Mondor Hospital, Paris-Est University, Inserm U955-IMRB-Team 18, 94010 Creteil, France

**Keywords:** hepatitis delta virus, seroprevalence, blood donors, hepatitis B virus, occult hepatitis B infection

## Abstract

Hepatitis delta virus (HDV) is an obligate satellite of hepatitis B virus (HBV). Dual HDV/HBV infection is associated with down-regulated HBV replication and fast progression to severe liver disease. Although HDV is transmissible through exposure to infected blood, data about HDV infection in blood donors remain scarce. Between 2011 and 2021, 869,633 donations were collected from prequalified donors in Dalian, China. In total, 1060 (0.12%) were confirmed HBsAg and/or HBV DNA-reactive. Subsequently, anti-HDV IgG was tested in 2175 donations, including 65 that tested HBsAg+ pre donation, 1017 confirmed HBV-positive (507 HBsAg+/HBV DNA+, 33 HBsAg+/DNA−, 477 HBsAg-/DNA+ (451 occult (OBI) and 26 acute infections)), 327 viral DNA non-repeated-reactive, 397 anti-HBc-only, and 369 anti-HBs-only. Two (0.09%) samples tested anti-HDV IgG weakly reactive but were unconfirmed by IgM and IgG repeat testing with alternative assays, suggesting an initial false reactivity. In addition, HDV testing in a subgroup of confirmed OBI donors, comprising 451 donors from Dalian and 126 archived samples of OBI donors from around the world, showed only one non-Chinese donor to be repeatedly anti-HDV-reactive, suggesting that HDV/HBV coinfection does not play a significant role in the genesis of OBI. The overall data suggested an extremely low prevalence of HDV infection among blood donors in Liaoning province, Northeast China.

## 1. Introduction

Hepatitis delta virus (HDV) is a defective blood-borne virus and an obligate satellite of hepatitis B virus (HBV). The HDV genome is a small single-stranded circular RNA (1700 nucleotides) that shares common features with plant viroids and contains a single open reading frame encoding the hepatitis delta antigen (HDAg) [1]. Two forms of the HDAg protein are produced after post-translational maturations to fulfill several functions in genome replication and the assembly of the nucleocapsid-like ribonucleoprotein (RNP) independently of HBV [2]. However, the assembly of infectious HDV virions and cell egress requires the coating of RNP with HBV envelope proteins. Consequently, HBV and HDV virions bear the same infectivity determinants and share a common tropism with human hepatocytes via interaction with the Human Sodium Taurocholate Co-transporting Polypeptide (NTCP). HDV/HBV dual infection occurs as a coinfection when the two viruses are acquired simultaneously, or as a superinfection when an HBV-infected individual acquires HDV secondarily [1]. The transmission of HDV is similar to that of HBV, i.e., blood, sexual, percutaneous, and, in rare cases, perinatal [3]. Acute simultaneous HDV and HBV infections can lead to mild-to-severe hepatitis with signs and symptoms indistinguishable from those of other types of acute viral hepatitis infections. Recovery is usually complete, the development of fulminant hepatitis is infrequent, and chronic hepatitis D is rare (less than 5% of acute hepatitis cases). However, HBV/HDV coinfection or superinfection is more likely to cause more severe chronic viral hepatitis with a higher and more rapid progression rate to cirrhosis and hepatocellular carcinoma (HCC) compared to HBV mono-infection [4,5]. Half of patients with chronic HDV/HBV infection have been reported to rapidly progress to cirrhosis within 5 years and to HCC within 10 years [5]. However, the mechanism by which HDV causes more severe hepatitis and a faster progression of fibrosis than HBV alone remains unclear. Recently, efforts have been made to develop specific antiviral treatments for HDV [6,7]. In addition, global HBV vaccination campaigns, especially among newborns, remain crucial to the eradication of HDV infection.

Because of the risk of a more severe form of chronic viral hepatitis associated with dual HDV/HBV infection, some guidelines recommend routine screening for HDV in HBV surface antigen (HBsAg) carriers to document the prevalence of HDV/HBV co- or superinfection, and to improve the clinical management of dually infected patients [8]. HDV diagnosis includes measuring total IgG to HDV (anti-HD) with validated commercially available enzyme immunoassays with sufficient specificity and sensitivity. IgM-specific assays are also available to detect primary infection. Active infection is confirmed by the detection of HDV RNA in blood by RT-PCR [9].

The global epidemiology of hepatitis D still remains uncertain because of the lack of recent and accurate epidemiological studies. The latest statements from the World Health Organization (WHO) indicate that HDV globally affects about 5% of chronic hepatitis B carriers [10]. According to multiple meta-analyses, the rate of HDV infection among HBV chronic carriers is estimated to range between 4.5% and 14.6% worldwide [5,11,12]. This uncertainty about HDV’s prevalence is related to several limitations, including the fact that most studies target selective, non-representative populations, primarily patients with clinically advanced HBV infection. There is also a need for improved, standardized, and widely available serological and molecular assays, as current in-house or commercial assays may have suboptimal sensitivity to detect all HDV genotypes, particularly those originating in Africa [13].

Although both HDV and HBV are transmissible by exposure to infected blood, blood donors dually infected with HDV and HBV, whether HBsAg-positive or HBsAg-negative, remain overlooked. Recently, a Chinese multicenter study reported low HDV seroprevalence among HBV-coinfected donors in 14 provinces or municipalities [14], but no data were available for Liaoning province in northeastern China. The objective of this study was firstly to document the prevalence of HDV among asymptomatic HBV-infected blood donors in Dalian, Liaoning Province. Second, HDV infection is a well-known factor in the worsening of chronic viral hepatitis, and it seemed of interest to study its potential association with a particular form of persistent low-level HBV infection, known as occult HBV infection (OBI). OBI is defined by the presence of replication-competent HBV DNA in the liver and/or HBV DNA in the blood of persons testing HBsAg-negative by the currently available assays with or without detectable anti-HBc or anti-HBs [15]. For this reason, the prevalence of HDV was also studied in a subgroup of blood donors with confirmed OBI, which included donors from Dalian as well as previously characterized OBI donors from other geographical areas.

## 2. Materials and Methods

### 2.1. Blood Donation HBV Screening

In Dalian Blood Center and collection sites, the general health and risk behaviors of candidate donors were assessed through a questionnaire and basic physical examination by a dedicated clinician. Eligible donors were tested pre donation for blood type, hemoglobin and alanine-aminotransferase (ALT) levels, and HBsAg using a rapid test (HBsAg Rapid test, InTec Products, Xiamen, China; 95% limit of detection (LoD): 5 IU/mL). Blood donations were collected from prequalified donors and further tested for HBsAg, anti-HCV, anti-HIV alone or in combination with HIV antigen, and antibodies against Treponema pallidum using two enzyme-linked immunosorbent assays (EIAs) for each marker, and for HBV DNA, HCV RNA, and HIV-1 RNA with multiplex nucleic acid testing (NAT) assays, as previously described [16].

The study was conducted according to the ethical guidelines of the 1975 Declaration of Helsinki and was approved by the Academic and Ethics Committee of Dalian Blood Center (N° LL-2020-14). All donors provided signed inform consent for their blood to be used for clinical or research purposes in relation to blood safety. Written consent forms are kept in Dalian Blood Center.

### 2.2. HBV Confirmatory Testing

Confirmation strategies for HBsAg and HBV DNA in blood donations have been described previously [16,17]. Briefly, plasma samples that tested repeat reactive (RR) for both HBsAg and HBV DNA were considered definitively infected with HBV without further confirmatory testing. HBsAg reactivity was confirmed in HBsAg+/HBV DNA− samples using an alternative electrochemiluminescence immunoassay (Elecsys HBsAg II; Roche Diagnostics, Manheim, Germany). HBsAg−/HBV DNA+ samples were considered truly reactive when viral DNA was confirmed reactive by use of an alternative nucleic acid test at the index donation or during follow-up. HBV DNA unconfirmed samples were considered as NAT non-discriminatory-reactive (NDR). A subset of pre-donation samples that showed reactivity to HBsAg with the rapid test were tested with the Elecsys HBsAg II assay, and for HBV DNA with in-house quantitative real-time PCR and/or nested PCR as previously described [17].

All samples confirmed HBsAg-reactive and/or HBV DNA-reactive were tested for both antibodies to anti-HBc and antibodies to HBV surface antigen (anti-HBs) using Elecsys Anti-HBc and Elecsys Anti-HBs II (Roche Diagnostics) assays.

### 2.3. Sample Selection

**Figure 1:**
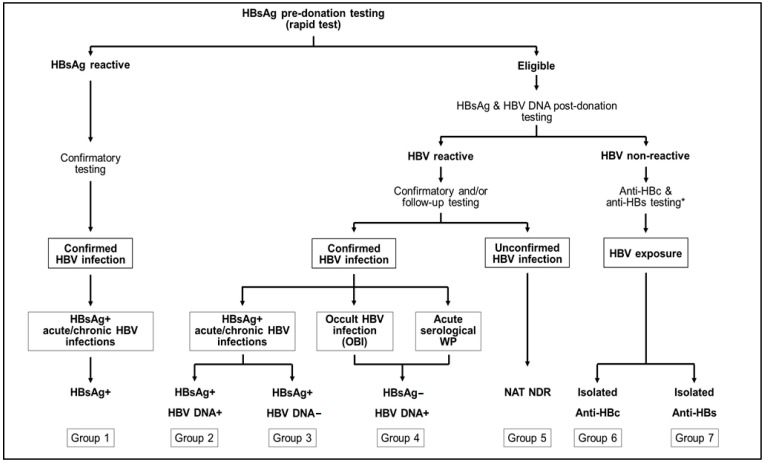
Schematic representation of the HBV testing and confirmation algorithms implemented at Dalian Blood Center. Seven groups of donors were identified with confirmed HBV infection pre or post donation (groups 1–4), suspected but unconfirmed HBV infection (group 5), or potential HBV exposure with no replicative infection (groups 6 and 7). * Testing performed as part of specific retrospective studies.

Samples included in the study were randomly selected from a repository of archived Dalian donor samples, which were classified into seven groups based on HBV testing and confirmation results (Figure 1). Group 1 consisted of plasma samples collected in 2022 from ineligible candidate donors with confirmed HBsAg reactivity prior to donation. Groups 2 to 4 included samples collected between 2011 and 2021 from HBV-infected donors with confirmed HBsAg and/or HBV DNA reactivity. Donors with no detectable HBsAg and a NAT NDR result, for whom HBV infection could possibly be suspected but not confirmed, were classified as group 5. In addition, plasma samples from fully qualified donors with retrospective isolated anti-HBc or isolated anti-HBs reactivity were also included in the study. These were residual samples randomly collected in 2017–2018 from a previous study involving supplementary retrospective serological testing [18]. Samples were stored at −20 °C.

In addition, a subgroup of confirmed OBI donors was formed that included 451 donors from Dalian and 126 archived plasma samples from previously characterized OBI donors originating from around the world (Belgium *n* = 2, Denmark *n* = 1, South Korea *n* = 3, Poland *n* = 43, South Africa *n* = 54, Spain *n* = 12, and Switzerland *n* = 11). These archived samples were collected between 2007 and 2013 as part of previous studies conducted under the aegis of the Transfusion-transmitted Infectious Diseases Working Party of the International Society of Blood Transfusion, and have been stored at −80 °C [19,20,21,22].

### 2.4. HDV Serological and Molecular Testing

HDV IgG was tested using WANTAI HDV-IgG ELISA (WANTAI Biological Pharmacy Enterprise Co., Beijing, China) and IgM/IgG using LIAISON XL Murex Anti-HDV assay (DiaSorin, Saluggia, Italy). Reactive samples were retested twice with the same assay. Repeat-reactive samples were tested further for HDV IgG and HDV IgM using a different assay (Beijing Beier Bioengineering Co., Ltd., Beijing, China). Samples that tested reactive with two different assays were considered to be confirmed reactive for anti-HDV. Anti-HDV-reactive samples were tested for HDV RNA using the EurobioPlex HDV qRT-PCR assay (LoD: 100 IU/mL; Eurobio Scientific, Courtaboeuf, France).

## 3. Results

### 3.1. Characterization of HBV Infection

**Figure 2:**
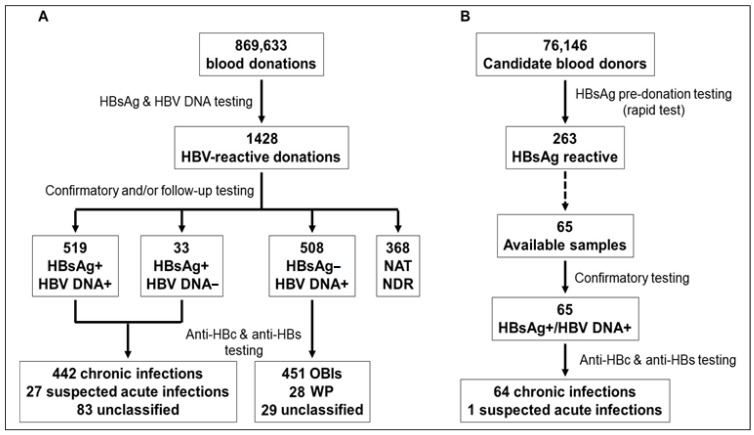
HBV screening and confirmatory testing among 869,633 prequalified blood donations collected between 2011 and 2021 (**A**), and 76,146 unqualified candidate donors after pre-donation testing in 2022 (**B**).

Between 2011 and 2021, 869,633 donations were collected from prequalified donors, of which 1060 (0.12%) were confirmed reactive for HBsAg and/or HBV DNA (Figure 2A). HBV-infected samples were distributed as follows: 519 HBsAg+/HBV DNA+ (0.06%), 33 HBsAg+/HBV DNA− (0.004%), and 508 HBsAg−/HBV DNA+ (0.06%). Additional serologic testing of 469 HBsAg+ donations identified 442 anti-HBc+ and/or anti-HBs+ donations, which were classified as chronic HBV infection regardless of HBV DNA reactivity. Twenty-seven samples with only HBsAg reactivity were suspected of acute HBV infection. A definitive HBV infection status could not be determined for 83 donations in the absence of additional anti-HBc/anti-HBs testing. Confirmatory and follow-up testing of 508 HBsAg−/HBV DNA+ donations identified 451 (88.8%) OBIs and 28 (5.5%) acute window period infections, whereas 29 (5.7%) samples with isolated HBV DNA reactivity could not be classified without follow-up. Three hundred and sixty-eight NAT-yield donations with unconfirmed HBV DNA reactivity were classified as NAT NDR and were further broken down as follows: 63 (17.1%) without HBV markers, 246 (66.8%) anti-HBc+, 56 (15.3%) anti-HBs only, and 3 (0.8%) not tested.

In 2022, pre-donation screening identified 263 (0.35%) HBsAg-reactive donors out of 76,146 ineligible candidate donors (Figure 2B). Serum samples were collected from only 65 of these HBsAg+ donors and included in the study. Additional testing identified 64 HBV chronic carriers and 1 suspected acute infection.

### 3.2. Characteristics of Enrolled Donors

**Figure 3:**
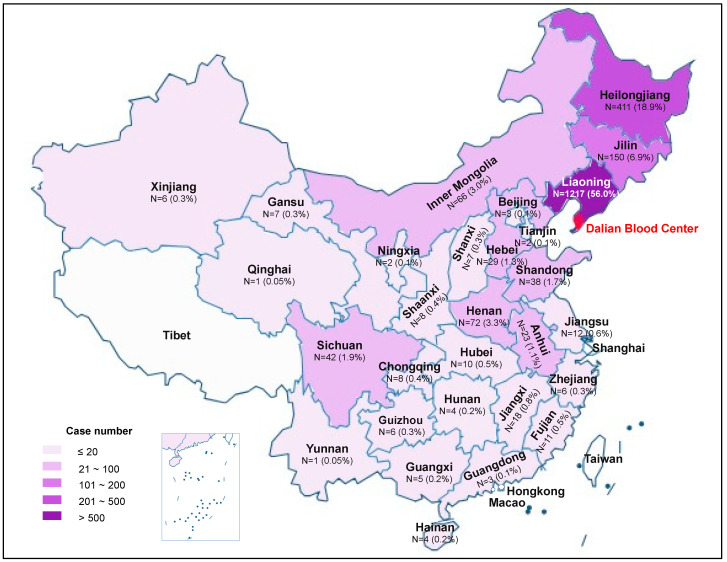
Distribution and geographic origins of 2175 candidate blood donors enrolled in the study and tested for HDV at Dalian Blood Center. The map was developed online at the official website of Standard Map Service System of China (http://bzdt.ch.mnr.gov.cn/index.html (accessed on 18 June 2023)).

Anonymous personal demographic information was collected from 2175 blood donors who were enrolled in the study (Table 1). Of the donors tested for HDV, 63.4% were male, 58.3% were first-time donors, and the median age was 37 years (range: 18–63) at the time of sample collection. Nearly 80% of donors were born before 1992, when the national program of neonatal hepatitis B vaccination in newborns was implemented. The majority (70.8%) of these individuals were born in rural areas according to information on personal identification cards. Overall, 56% percent of the donors are from Liaoning province, where Dalian is located, while 44% of the donors moved to Dalian from 29 provinces, municipalities, and autonomous regions either permanently or temporarily (Figure 3). Heilongjiang province was the largest source of donors (18.9%) apart from Liaoning province; Jilin province was the second largest (6.9%), followed by Henan province (3.3%) and Inner Mongolia (3.0%).

### 3.3. HDV Detection

A total of 2175 Dalian blood donor samples were tested for anti-HDV antibodies, including 65/263 (24.7%) pre-donation HBsAg-reactive samples; 1017 samples confirmed HBV-infected after donation, including 507/519 (97.7%) HBsAg+/HBV DNA+, 33/33 (100%) HBsAg+/HBV DNA-, and 477/508 (93.9%) HBsAg−/HBV DNA+ (451 OBIs and 26 WP infections); 327/368 (88.9%) NAT NDR samples; and 766 samples comprising 397 anti-HBc+-only and 369 anti-HBs+-only qualified donations (Table 1 and Table 2). Two samples (0.09%) from a confirmed anti-HBc+/anti-HBs+ OBI donor and an anti-HBs-only eligible donor tested weakly reactive for anti-HDV IgG (sample/cut-off (S/CO) values: 2.3 and 1.8). Repeat testing and alternative IgM and IgG tests were performed on these samples but none were reactive, suggesting an initial false reactivity.

### 3.4. HDV and Occult HBV Infection

To test the hypothesis that HDV infection may contribute to the genesis of OBI, anti-HDV was tested in the subgroup of 451 confirmed OBI donors from Dalian infected with HBV genotype B and genotype C strains (Table 3). In addition, we tested 126 plasma samples from non-Chinese blood donors with previously characterized OBI who were infected with HBV genotypes A1, A2, and D strains (Table 3). Out of these 577 OBI donors, only one (0.17%) sample was repeatedly anti-HDV reactive (10 arbitrary units/mL; LIAISON XL Murex Anti-HDV). The corresponding donor originated from Poland and was infected with HBV genotype D. No HDV RNA was detected in the plasma, suggesting a past recovered infection.

## 4. Discussion

Although known for decades, HDV infection has been rather neglected in viral hepatitis. Recently, HDV infection has received renewed attention worldwide, likely due to the WHO’s global viral hepatitis strategy, which aims to reduce new hepatitis infections by 90% by 2030, and the recent development of HDV-specific antiviral treatments [8,10]. Nevertheless, the regional, national, and global epidemiology of HDV remains poorly documented, although recent reports have suggested changing patterns [13,23]. Differences between selective and non-representative study populations, the often-limited number of samples included in studies, and the limited availability and standardization of the anti-HDV and HDV-RNA tests used can lead to major discrepancies in data that contribute to the global uncertainty around HDV prevalence [13]. Despite a significant decrease in HBsAg prevalence in recent decades, China remains a highly endemic country for HBV with an overall HBsAg prevalence of 6.5% and an estimated 1,000,000 newly diagnosed chronic infections in 2021, creating the conditions for sustained HDV infection [11,24]. However, a low incidence rate of HDV infection, varying between 0.03/100,000 in 2016 and 0.0177/100,000 in 2021, has been reported in Chinese patients by the National Health Commission of the People’s Republic of China (http://www.nhc.gov.cn (accessed on 18 June 2023)).

Blood donors appear as an alternative for assessing the epidemiology of HDV as they represent a homogenous group routinely tested for HBV markers with standardized methods, although they do not truly represent the general population. A recent study reported an overall seroprevalence of HDV of 0.067% among HBsAg-positive donors from mainland China, which translated into an extremely low prevalence (<1/1,000,000) among all blood donors [14]. However, donors originated from only 14 provinces and may not be representative of the whole country. Indeed, no data were available for the northeastern Liaoning province. In the present study, the retrospective screening of 2175 blood donations collected over 10 years in Dalian, Liaoning, showed no HDV seroreactivity in blood donors with confirmed or suspected markers of HBV infection.

The consistently low HDV prevalence observed among blood donors in the present study and in the study by Chang et al. [14] was markedly different from a 0.45% estimate reported for the general Chinese population in a recent meta-analysis [11]. This discrepancy may be linked to differences between the populations studied. Blood donors constitute a selected healthy population that does not truly reflect the general population. In contrast, patients with liver disease were included in the meta-analysis. Clinically driven HDV testing may introduce a bias, since it has been shown that the probability of finding anti-HDV was 4–10 times higher in patients with severe liver disease than in blood donors [25,26]. Furthermore, the 0.45% estimated HDV prevalence in China was extrapolated from a meta-analysis that included data from five studies conducted in mainland China, but with no indication about the exact geographic location. Yet it has been reported that HDV was unevenly distributed among HBsAg carriers in China and was present in hotspots [14,25]. Indeed, the estimated seroprevalence of HDV among Chinese individuals ranged between 0.00% and 0.62% in the study by Chen et al. [11]. Similarly, no anti-HDV reactivity was observed in 2634 samples from HBsAg+ patients admitted to hospitals who originated from seven provinces including Liaoning [25]. The variability in the serological markers used to define HDV infection and the difference in assay performance, as well as the absence of confirmatory testing, could also lead to an overestimation of HDV seroprevalence. A risk of false positivity was suggested in the present study by two samples that were initially weakly reactive and not subsequently confirmed.

The data of the present study suggested an extremely low risk of exposure to HDV among Dalian blood donors. As mentioned above, HDV infection in HBsAg+ patients seemed limited to geographical hotspots, in particular Inner Mongolia and Xinjiang [14,25]. Only 3% (*n* = 66) and 0.3% (*n* = 6) of Dalian donors came from Inner Mongolia and the western province of Xinjiang, respectively. Several factors may explain the apparent absence of HDV infection, including the limited number of samples studied with confirmed active HBV infection (*n* = 1017), along with the relatively low prevalence of HBV (0.12%) in healthy blood donors, which differed significantly from the 6.5% HBsAg+ figure reported in the general Chinese population. Pre-donation rapid testing also prevents people with high levels of ALT (≥50 U/L) and/or who are reactive to HBsAg from donating blood. To limit this potential population bias, rapid-test HBsAg-positive donor candidates were taken into account in this study, but only 65 (24.7%) samples could be included. NAT NDR samples (327/368; 88.9%) were also included, as it has previously been shown that HBV DNA carriage can be confirmed by extended testing in around 66% of cases [17]. In addition, underscreening for HDV markers in HBV carriers with no detectable HBsAg may lead to a significant underestimation of the global HDV prevalence [27].

Previous studies, albeit limited, have reported an HDV seroprevalence of approximately 4% in selected individuals reactive to HBV anti-core antibodies (anti-HBc) as a single serologic marker of HBV infection [28,29]. Therefore, donors with anti-HBc as the only marker of HBV exposure were investigated. Anti-HBs-only donors should be considered as a particular subgroup as anti-HBs may be more likely to originate from HBV vaccination rather than viral infection. However, an isolated anti-HBs serological profile has been reported in 8–13% of Chinese donors with transient or persistent OBI [30]. Extending recruitment to individuals not only confirmed to be carriers of HBV infection but also to those with only signs of exposure to the virus theoretically limited the risk of underestimating HDV prevalence. The poor performance of serological tests may be responsible for the false-negative results. This was unlikely because different ELISA tests were used for primary and confirmatory screening, with a clinical sensitivity of 100% according to the manufacturers, and anti-HBV IgG is known to persist for months after coinfection or superinfection, even after clearance of the virus [31].

The implementation of highly sensitive and effective testing algorithms to detect HBV in blood donations significantly reduced the HBV transfusion-transmission risk, and should therefore theoretically also limit the risk of HDV transmission. The HBV residual risk is mainly associated with rare cases of undetected OBI with HBV DNA loads below the detection threshold of the currently most sensitive NAT assays. However, HDV replication has been associated with HBV repression in animal and in vitro infection models, and with the suppression or fluctuation of HBV DNA and/or HBsAg in both the liver and serum of superinfected HBV chronic carriers [32,33,34,35]. Indeed, HDV infection has been reported in asymptomatic individuals with OBI and, as mentioned above, in individuals with isolated anti-HBc reactivity [28,29,36]. It was therefore tempting to hypothesize that HDV infection may contribute to this viral hepatitis residual transfusion risk by being a factor in barely detectable low-level OBI. However, the data obtained from 577 donors of various geographical origins showed a seropositivity rate of 0.17% (1/577) with no detectable viral RNA, and do not seem to support this hypothesis, nor do they argue in favor of an active role of HDV infection in the genesis of OBI. Recently, there has been concern that HDV infection can be spread and ultimately transmitted from human to human in the absence of HBV by unrelated enveloped viruses, i.e., HCV [37]. However, the impact of these mainly in vitro findings appeared very likely to be limited in clinical terms and not relevant to blood safety [24,38].

In conclusion, the retrospective screening of 2175 samples collected over a 10-year period revealed no HDV seroreactivity in blood donors with confirmed HBV infection or suspected HBV exposure in Dalian, Liaoning province. These results confirmed the extremely low prevalence of HDV in Chinese blood donors and a low risk of HDV transmission through blood transfusion. In addition, HDV/HBV coinfection appears to play no significant role in the genesis of OBI.

## Figures and Tables

**Table 1:** Characteristics of 2175 enrolled donors.

Parameters	Total	Pre-Donation Testing	Confirmed HBV Infections			NAT NDR	Qualified Donations	
		HBsAg+ (Group 1)	HBsAg+ HBV DNA+ (Group 2)	HBsAg+ HBV DNA− (Group 3)	HBsAg− HBV DNA+ (Group 4)	HBsAg− NAT +/− (Group 5)	Isolated Anti-HBc+ (Group 6)	Isolated Anti-HBs+ (Group 7)
N	2175	65	507	33	477*	327	397	369
Gender								
Male	1379	56	330	19	364	218	232	207
	(63.4%)	(86.2%)	(65.1%)	(57.6%)	(76.3%)	(66.7%)	(58.4%)	(56.1%)
Female	796	9	177	14	113	109	165	162
	(36.6%)	(13.8%)	(34.9%)	(42.4%)	(23.7%)	(33.3%)	(41.6%)	(43.9%)
Median age (year)	37 (18–63)	42 (18–54)	37 (18–59)	35 (19–56)	43 (18–60)	41 (18–63)	39 (18–59)	26 (18–57)
Year range								
18–25	473	13	126	7	61	56	30	180
	(21.7%)	(20.0%)	(24.9%)	(21.2%)	(12.8%)	(17.1%)	(7.6%)	(48.8%)
26–35	517	12	108	12	75	61	119	130
	(23.8%)	(18.4%)	(21.3%)	(36.4%)	(15.7%)	(18.7%)	(30.0%)	(35.2%)
36–45	592	21	142	10	147	93	142	37
	(27.2%)	(32.3%)	(28.0%)	(30.3%)	(30.8%)	(28.4%)	(35.8%)	(10.0%)
46–55	543	19	124	3	177	101	99	20
	(25.0%)	(29.2%)	(24.5%)	(9.1%)	(37.1%)	(30.9%)	(24.9%)	(5.4%)
>55	50	0	7	1	17	16	7	2
	(2.3%)		(1.4%)	(3.0%)	(3.6%)	(4.9%)	(1.8%)	(0.5%)
Birth year								
<1992	1763	47	424	29	434	279	365	185
	(81.0%)	(72.3%)	(83.6%)	(87.9%)	(91.0%)	(85.3%)	(91.9%)	(50.1%)
≥1992	412	18	83	4	43	48	32	184
	(19.0%)	(27.7%)	(16.4%)	(12.1%)	(9.0%)	(14.7%)	(8.1%)	(49.9%)
Donor type								
First donor	1268	63	496	29	234	131	145	170
	(58.3%)	(96.9%)	(97.8%)	(87.9%)	(49.1%)	(40.0%)	(36.5%)	(46.7%)
Repeat donor	907	2	11	4	243	196	252	199
	(41.7%)	(3.1%)	(2.2%)	(12.1%)	(50.9%)	(60.0%)	(63.5%)	(53.3%)
Originating province								
Liaoning	1217	29	244	19	266	191	246	222
	(56.0%)	(44.6%)	(48.1%)	(57.6%)	(55.8%)	(58.4%)	(62.0%)	(60.2%)
Heilongjiang	411	19	93	9	89	66	77	58
	(18.9%)	(29.2%)	(18.3%)	(27.3%)	(18.7%)	(20.2%)	(19.4%)	(15.7%)
Jilin	150	7	41	0	37	17	25	23
	(6.9%)	(10.8%)	(8.1%)		(7.8%)	(5.2%)	(6.3%)	(6.2%)
Henan	72	2	17	1	13	9	11	19
	(3.3%)	(3.1%)	(3.4%)	(3.0%)	(2.7%)	(2.8%)	(2.8%)	(5.1%)
Inner Mongolia	66	5	16	0	18	8	7	12
	(3.0%)	(7.7%)	(3.2%)		(3.8%)	(2.4%)	(1.8%)	(3.3%)
Sichuan	42	0	21	2	9	5	2	1
	(1.9%)		(4.1%)	(6.1%)	(1.9%)	(1.5%)	(0.5%)	(0.3%)
Shandong	38	2	8	0	9	7	7	7
	(1.7%)	(3.1%)	(1.6%)		(1.9%)	(2.1%)	(1.8%)	(1.9%)
Hebei	29	0	7	0	10	3	1	8
	(1.3%)		(1.4%)		(2.1%)	(0.9%)	(0.3%)	(2.2%)
Anhui	23	0	7	0	6	1	4	5
	(1.1%)		(1.4%)		(1.3%)	(0.1%)	(1.0%)	(1.4%)
Others	127	1	57	2	20	20	17	14
	(5.8%)	(1.5%)	(11.2%)	(6.1%)	(4.2%)	(6.1%)	(4.3%)	(3.8%)
Original registration setting								
Urban	589	13	99	15	131	109	11	108
	(27.1%)	(20.0%)	(19.5%)	(45.5%)	(27.5%)	(33.3%)	(28.7%)	(29.3%)
Rural	1540	45	388	18	339	216	279	255
	(70.8%)	(69.2%)	(76.5%)	(54.5%)	(71.1%)	(66.1%)	(70.3%)	(69.1%)
Not specified	46	7	20	0	7	2	4	6
	(2.1%)	(10.8%)	(3.9%)		(1.5%)	(0.6%)	(1.0%)	(1.6%)

**Table 2:** Fractions of categorized blood donors tested for anti-HDV.

Sample Categories	Total Number of Samples Tested for HBV	Number of Samples Tested for Anti-HDV (%)
Pre-donation testing		
HBsAg+ (Group 1)	263	65 (24.7%)
Post-donation testing		
HBsAg+/HBV DNA+ (Group 2)	519	507 (97.7%)
HBsAg+/HBV DNA− (Group 3)	33	33 (100%)
HBsAg−/HBV DNA+ (Group 4)	508	477 (93.9%) *
HBsAg−/NAT NDR (Group 5)	368	327 (88.9%)
Isolated anti-HBc (Group 6)	397	397 (100%)
Isolated anti-HBs (Group 7)	369	369 (100%)
Total	2457	2175 (88.5%)

* 451 confirmed OBIs and 26 acute infections.

**Table 3:** Anti-HDV testing of 577 blood donors with OBI.

Countries	N Anti-HDV Reactive/ N Tested	N HBV Sequences	HBV Genotypes				
			A1	A2	B	C	D
China	0/451	151			23	125	3
		(34%)			(15%)	(83%)	(2%)
Belgium	0/2	2					2
		(100%)					(100%)
Denmark	0/1	1					1
		(100%)					(100%)
Poland	1/43	21		9			12
	(2.3%)	(49%)		(43%)			(57%)
South Africa	0/54	36	29				7
		(67%)	(81%)				(19%)
South Korea	0/3	3				3	
		(100%)				(100%)	
Spain	0/12	12		3			9
		(100%)		(25%)			(75%)
Switzerland	0/11	7					7
		(64%)					(100%)
Total	1/577	233	29	12	23	128	41
	(0.17%)	(40%)	(12%)	(5%)	(10%)	(55%)	(18%)

## Data Availability

The data presented in this study are available on request from the corresponding author.

## References

[1] Urban S., Neumann-Haefelin C., Lampertico P. (2021). Hepatitis D virus in 2021: Virology, immunology and new treatment approaches for a difficult-to-treat disease. Gut.

[2] Sureau C., Negro F. (2016). The hepatitis delta virus: Replication and pathogenesis. J. Hepatol..

[3] Sellier P.O., Maylin S., Brichler S., Berçot B., Lopes A., Chopin D., Pogliaghi M., Munier A.L., Delcey V., Simoneau G. (2018). Hepatitis B Virus-Hepatitis D Virus mother-to-child co-transmission: A retrospective study in a developed country. Liver Int..

[4] Fattovich G., Giustina G., Christensen E., Pantalena M., Zagni I., Realdi G., Schalm S.W., The European concerted action on viral hepatitis (Eurohep) (2000). Influence of hepatitis delta virus infection on morbidity and mortality in compensated cirrhosis type B. Gut.

[5] Miao Z., Zhang S., Ou X., Li S., Ma Z., Wang W., Peppelenbosch M.P., Liu J., Pan Q. (2020). Estimating the global prevalence, disease progression, and clinical outcome of hepatitis delta virus infection. J. Infect. Dis..

[6] Yardeni D., Heller T., Koh C. (2022). Chronic hepatitis D-What is changing?. J. Viral Hepat..

[7] Lampertico P., Roulot D., Wedemeyer H. (2022). Bulevirtide with or without pegIFNα for patients with compensated chronic hepatitis delta: From clinical trials to real-world studies. J. Hepatol..

[8] Hughes S.A., Wedemeyer H., Harrison P.M. (2011). Hepatitis delta virus. Lancet.

[9] Olivero A., Smedile A. (2012). Hepatitis delta virus diagnosis. Semin. Liver Dis..

[10] World Health Organization World Hepatitis Summit 2022 Statement. https://www.who.int/news/item/10-06-2022-world-hepatitis-summit-2022-statement.

[11] Chen H.Y., Shen D.T., Ji D.Z., Han P.C., Zhang W.M., Ma J.F., Chen W.S., Goyal H., Pan S., Xu H.G. (2019). Prevalence and burden of hepatitis D virus infection in the global population: A systematic review and meta-analysis. Gut.

[12] Stockdale A.J., Kreuels B., Henrion M.Y.R., Giorgi E., Kyomuhangi I., de Martel C., Hutin Y., Geretti A.M. (2020). The global prevalence of hepatitis D virus infection: Systematic review and meta-analysis. J. Hepatol..

[13] Rizzetto M., Hamid S., Negro F. (2021). The changing context of hepatitis D. J. Hepatol..

[14] Chang L., Yan Y., Ji H., Sun H., Jiang X., Lu Z., Wang L. (2022). and HBV-Infected Blood Donors Study Group. Low seroprevalence of hepatitis delta virus co-infection in hepatitis B virus-infected blood donors in China: A multicenter study. Front. Microbiol..

[15] Raimondo G., Locarnini S., Pollicino T., Levrero M., Zoulim F., Lok A.S., Taormina Workshop on Occult HBV Infection Faculty Members (2019). Update of the statements on biology and clinical impact of occult hepatitis B virus infection. J. Hepatol..

[16] Deng X., Zang L., Wang X., Chen H., Liu J., Gao Y., Xu S., Wang L., Fan Y., Candotti D. (2020). Follow-up program for blood donors with unconfirmed screening results reveals a high false-positive rate in Dalian, China. Transfusion.

[17] Deng X., Guo X., Li T., Laperche S., Zang L., Candotti D. (2022). Alternative hepatitis B virus DNA confirmatory algorithm identified occult hepatitis B virus infection in Chinese blood donors with non-discriminatory nucleic acid testing. Blood Transfus..

[18] Zhou L., Wang D., Zang L., Deng X. (2023). A follow-up study of blood donors with HBV core antibody. Chin. J. Blood Transfus..

[19] Candotti D., Grabarczyk P., Ghiazza P., Roig R., Casamitjana N., Iudicone P., Schmidt M., Bird A., Crookes R., Brojer E. (2008). Characterization of occult hepatitis B virus from blood donors carrying genotype A2 or genotype D strains. J. Hepatol..

[20] Allain J.P., Belkhiri D., Vermeulen M., Crookes R., Cable R., Amiri A., Reddy R., Bird A., Candotti D. (2009). Characterization of occult hepatitis B virus strains in South African blood donors. Hepatology.

[21] Candotti D., Lin C.K., Belkhiri D., Sakuldamrongpanich T., Biswas S., Lin S., Teo D., Ayob Y., Allain J.P. (2012). Occult hepatitis B infection in blood donors from South East Asia: Molecular characterisation and potential mechanisms of occurrence. Gut.

[22] Allain J.P., Mihaljevic I., Gonzalez-Fraile M.I., Gubbe K., Holm-Harritshøj L., Garcia J.M., Brojer E., Erikstrup C., Saniewski M., Wernish L. (2013). Infectivity of blood products from donors with occult hepatitis B virus infection. Transfusion.

[23] Fallon B.S., Cooke E.M., Hesterman M.C., Norseth J.S., Akhundjanov S.B., Weller M.L. (2023). A changing landscape: Tracking and analysis of the international HDV epidemiology 1999–2020. PLoS Glob. Public Health.

[24] Razavi H.A., Buti M., Terrault N.A., Zeuzem S., Yurdaydin C., Tanaka J., Aghemo A., Akarca U.S., Al Masri N.M., Alalwan A.M. (2023). Hepatitis D double reflex testing of all hepatitis B carriers in low-HBV- and high-HBV/HDV-prevalence countries. J. Hepatol..

[25] Roggenbach I., Chi X., Lempp F.A., Qu B., Walter L., Wu R., Gao X., Schnitzler P., Ding Y., Urban S. (2021). HDV Seroprevalence in HBsAg-Positive Patients in China Occurs in Hotspots and Is Not Associated with HCV Mono-Infection. Viruses.

[26] Caviglia G.P., Ciancio A., Rizzetto M. (2022). A Review of HDV Infection. Viruses.

[27] Tharwani A., Hamid S. (2023). Elimination of HDV: Epidemiologic implications and public health perspectives. Liver Int..

[28] Genné D., Rossi I. (2011). Hepatitis delta in Switzerland: A silent epidemic. Swiss Med. Wkly..

[29] Mhalla S., Kadri Y., Alibi S., Letaief A., Boukadida J., Hannachi N. (2016). Hepatitis D Virus Infection Among Hepatitis B Surface Antigen Carriers and in “Isolated anti-HBc” Antibodies Profile in Central Tunisia. Hepat. Mon..

[30] Deng X., Guo X., Gu H., Wang D., Laperche S., Allain J.P., Zang L., Candotti D. (2022). Anti-HBc-nonreactive occult hepatitis B infections with HBV genotypes B and C in vaccinated immunocompetent adults. J. Viral. Hepat..

[31] Chen L.Y., Pang X.Y., Goyal H., Yang R.X., Xu H.G. (2021). Hepatitis D: Challenges in the estimation of true prevalence and laboratory diagnosis. Gut Pathog..

[32] Pastore G., Monno L., Santantonio T., Angarano G., Milella M., Giannelli A., Fiore J.R. (1990). Hepatitis B virus clearance from serum and liver after acute hepatitis delta virus superinfection in chronic HBsAg carriers. J. Med. Virol..

[33] Wu J.C., Chen P.J., Kuo M.Y., Lee S.D., Chen D.S., Ting L.P. (1991). Production of hepatitis delta virus and suppression of helper hepatitis B virus in a human hepatoma cell line. J. Virol..

[34] Schaper M., Rodriguez-Frias F., Jardi R., Tabernero D., Homs M., Ruiz G., Quer J., Esteban R., Buti M. (2010). Quantitative longitudinal evaluations of hepatitis delta virus RNA and hepatitis B virus DNA shows a dynamic, complex replicative profile in chronic hepatitis B and D. J. Hepatol..

[35] Alfaiate D., Lucifora J., Abeywickrama-Samarakoon N., Michelet M., Testoni B., Cortay J.C., Sureau C., Zoulim F., Dény P., Durantel D. (2016). HDV RNA replication is associated with HBV repression and interferon-stimulated genes induction in super-infected hepatocytes. Antiviral Res..

[36] Delfino C.M., Eirin M.E., Berini C., Malan R., Gentile E., Castillo A., Pedrozo W., Krupp R., Blejer J., Oubiña J.R. (2012). HDAg-L variants in covert hepatitis D and HBV occult infection among Amerindians of Argentina: New insights. J. Clin. Virol..

[37] Perez-Vargas J., Amirache F., Boson B., Mialon C., Freitas N., Sureau C., Fusil F., Cosset F.L. (2019). Enveloped viruses distinct from HBV induce dissemination of hepatitis D virus in vivo. Nat. Commun..

[38] Cappy P., Lucas Q., Kankarafou N., Sureau C., Laperche S. (2021). No Evidence of Hepatitis C Virus (HCV)-Assisted Hepatitis D Virus Propagation in a Large Cohort of HCV-Positive Blood Donors. J. Infect. Dis..

